# A Weighted Neural Network Model Based on Laboratory Tests for Identifying Lymph Node Metastases in Esophageal Squamous Cell Carcinomas

**DOI:** 10.3390/bios16070377

**Published:** 2026-07-10

**Authors:** Qiangqiang Ouyang, Ziming Gao, Jingbo Yang, Shaoyi Wang, Zonglin Li, Yifan Zhang, Tianyou Chen, Xinhua Xu, Runkun Han, Hao Chen

**Affiliations:** 1 College of Artificial Intelligence and Low-Altitude Technology, South China Agricultural University, Guangzhou 510642, China; oyqq@scau.edu.cn (Q.O.); jingboyang@cuhk.edu.hk (J.Y.); lizonglin@stu.scau.edu.cn (Z.L.);; 2 Ultrasound Department, Guangdong Provincial Occupational Disease Prevention Hospital, Guangzhou 510300, China; 3 Department of Clinical Laboratory, State Key Laboratory of Oncology in South China, Guangdong Oesophageal Cancer Research Institute, Sun Yat-Sen University Cancer Center, Guangzhou 510060, China; xuxinh@sysucc.org.cn

**Keywords:** lymph node metastasis (LNM), esophageal squamous cell carcinoma (ESCC), weighted neural network (WNN), lymph node metastases, machine learning

## Abstract

Lymph node metastasis (LNM) is a key prognostic factor in esophageal squamous cell carcinoma (ESCC), and accurate preoperative prediction remains challenging. Blood biomarkers provide a conventional, preoperative diagnostic technique that is cost-effective and free from radiation risks. So far, previous studies have been published on the precise diagnosis of lymph node metastases using conventional ultrasound or CT techniques. While there is a lack of research studies that address the diagnosis of LNM from blood biomarkers. In this work, we acquired a cohort of blood biomarkers of 1933 patients and designed a weighted neural network (WNN) model for the accurate prediction of LNM from blood biomarkers. The WNN model is designed with a neural network classifier trained on blood biomarkers labeled with pathological nodal (pN) stages of LNM. The experimental findings demonstrate that the WNN model achieved 83.1% accuracy and an AUC of 0.88 on the original, non-augmented test set for diagnosing LNM, while CT only achieved 50.4% accuracy (AUC 0.60) and ultrasound achieved 60.5% accuracy (AUC 0.67). Additionally, SHAP analysis reveals that three blood biomarkers—white blood cells (WBC#), monocytes (Mono#), and neutrophils (Neut#)—significantly impact the WNN model’s output. This WNN model shows promise as a research tool to diagnose LNM.

## 1. Introduction

Esophageal cancer (EC) is one of the most aggressive malignancies worldwide and is the sixth leading cause of cancer-related mortality [1,2]. Histologically, esophageal cancer (EC) is classified into esophageal squamous cell carcinoma (ESCC), esophageal adenocarcinoma (EAC), and small cell carcinoma of the esophagus (SCCE) [3]. Around 90% of ECs globally are ESCC. Currently, the 5-year survival rate of ESCC patients is around 20% for regional disease and 40% for localized disease [4,5,6,7]. In respectable tumor patients, surgery is the mainstay of treatment. However, lymphadenectomy-induced complications and LNM complicate surgical decisions. The complications caused by lymph node (LN) resection and postoperative LNM should not be ignored since they cause 5–10% mortality and 50% incidence rate after surgery [8]. Theoretically, removing a greater number of suspicious lymph nodes could lead to increased benefits, including improved staging accuracy, reduced risk of recurrence after surgery, and higher rates of survival. However, the excessive resection of lymph nodes in close proximity to the esophagus may result in elevated rates of postoperative complications and mortality as a consequence of their distinct anatomical positioning. Hence, precise preoperative evaluation of LNM in EC is essential for clinicians in determining appropriate surgical interventions [9]. Accurate LN dissection plays a critical role in achieving optimal surgical outcomes while minimizing postoperative complications and mortality rates. Presently, a combination of endoscopy, esophagography, endoscopic ultrasonography, computed tomography (CT), and 18F-fluorodeoxyglucose positron emission tomography (PET) is utilized for the diagnosis of ESCC and the assessment of clinical stage cancer [10]. Nevertheless, these methods are insufficient for accurately determining the LN status in ESCC patients [11,12,13]. Although numerous molecular biomarkers for LNM have been discovered, most of these biomarkers exhibit restricted detection capabilities and are not readily applicable in clinical settings.

Routine laboratory items like hematological and biochemical tests are low-cost and widely utilized, proving valuable insight into the differences in various disease states [14,15,16]. Routine laboratory tests have been shown to be biomarkers useful for diagnosing and predicting the prognosis of ESCC. Parameters such as NLR and PLR in blood cell counts are proven indicators for predicting LNM in ESCC patients [15]. Right now, there is a deficiency in systematic research regarding the value of summarizing all parameters as biomarkers for LNM. Machine learning has become particularly useful for analyzing complex and heterogeneous clinical data types, using them to make medical-related predictions in recent years [17]. This retrospective study aimed to systematically assess the capability of laboratory tests in predicting LNM through machine learning methods. The resulting model is presented as a proof-of-concept requiring extensive prospective validation and clinical utility assessment before any clinical deployment.

**Figure 1:**
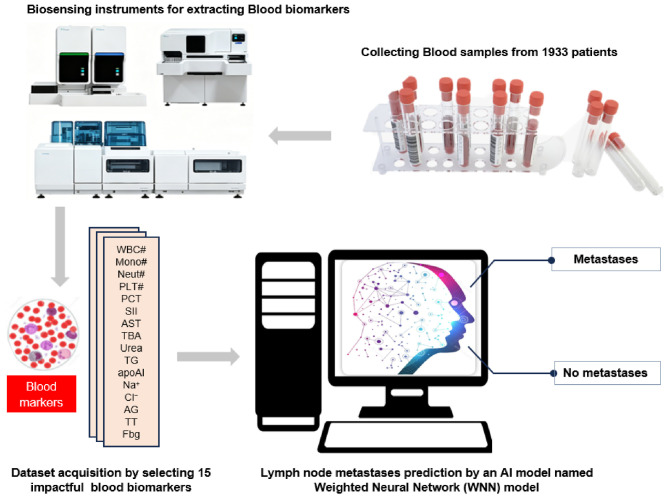
Concept of the neural network-based biosensing system for predicting lymph node metastases for a precision treatment strategy.

The workflow for predicting LNM using a combination of biochemical analysis and artificial intelligence is illustrated in Figure 1. Blood samples are collected from patients and processed using several biosensing instruments to extract relevant blood biomarkers such as WBC, PLT, and TG. These biomarkers are organized into datasets and input into an AI-based computational system. The AI algorithm analyzes the data, represented by the weighted neural network (WNN) model, to predict whether LNM is present. This workflow highlights the application of advanced biochemical testing and machine learning techniques in oncology diagnostics.

## 2. Results

### 2.1. Characteristics of Patients

We collected patients’ data from Sun Yat-sen University Cancer Center, Guangzhou (SYSUCC cohort, *n* = 1933). The clinical characteristics of patients in both the training and testing cohorts, along with their respective *p*-values, are shown in Table 1. The mean age of patients in the training cohort is 66.5 ± 8.5 years, while in the testing cohort, it is 64.3 ± 7.9 years. Gender distribution is similar between the cohorts, with the training cohort consisting of 890 males and 231 females, and the testing cohort having 622 males and 190 females (*p* = 0.294). Regarding clinical stage, the number of patients classified as pN0 is 622 in the training cohort and 520 in the testing cohort, whereas pN1-3 includes 499 and 292 cases, respectively.

### 2.2. The Analysis of Blood Biomarker Relates to Clinical Stage of LNM

**Figure 2:**
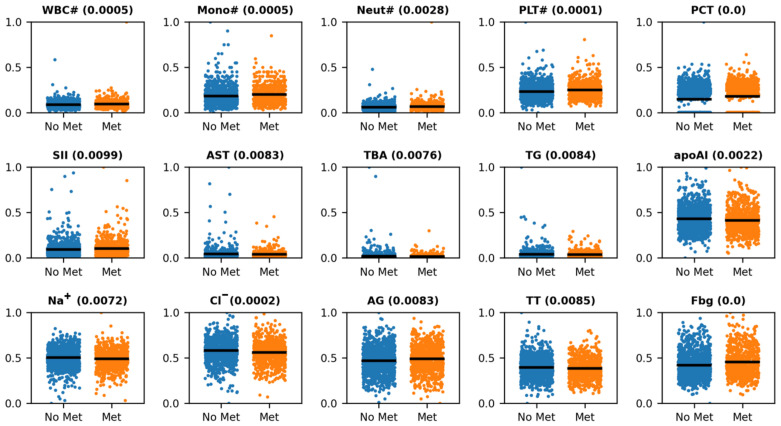
The selected blood biomarkers that have a significant impact on the pN stage of lymph node metastases with significance < 0.01. *p* in the brackets represents the *p*-value of the difference significance analysis between a biomarker value of metastases and one without metastases. The significance analysis is carried out by the Wilcoxon Rank Sum test for any of the biomarkers.

To analyze the relationship between blood biomarkers and the clinical stage of LNM, we present multiple box plots comparing biomarker levels between patients with metastases (“Met”) and those without metastases (“No Met”) as shown in Figure 2. The *p*-values in brackets indicate the statistical significance of differences between these groups, with all biomarkers shown having a significance level of *p* < 0.01, determined by the Wilcoxon Rank Sum test. Biomarkers such as WBC#, PLT#, PCT, and Cl- exhibit extremely low *p*-values (*p* = 0.0), suggesting strong differences between the metastatic and non-metastatic groups. Other biomarkers, including Mono#, Neut#, apoAI, and Na+, also show statistically significant differences (*p* < 0.01). These findings indicate that these blood biomarkers may be relevant for assessing the presence of LNM in patients.

**Figure 3:**
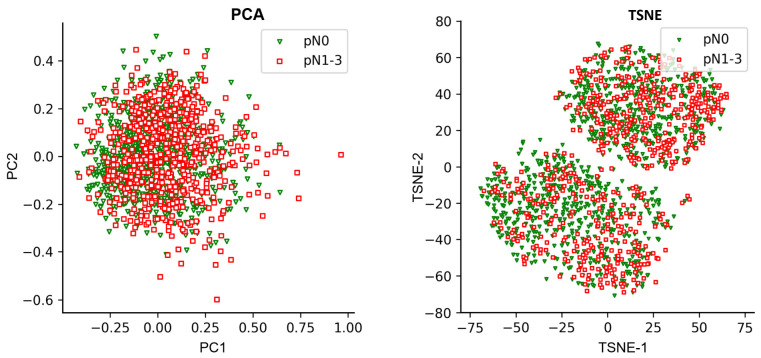
PCA (principal component analysis) and t-SNE analysis of blood biomarker data were used for the whole dataset, which shows their structural diversity and difficulties in identifying lymph node metastases.

To visualize the structural diversity of blood biomarker data and assess its effectiveness in distinguishing LNM, we present PCA (principal component analysis) and t-SNE (t-distributed stochastic neighbor embedding) scatter plots as shown in Figure 3. The left plot (PCA) and the right plot (t-SNE) display the distribution of biomarker data for patients with (pN1-3) and without (pN0) metastases in a two-dimensional space. The overlapping distributions in both plots indicate that the biomarker data for these groups are not well-separated, suggesting difficulties in distinguishing between metastatic and non-metastatic cases based on these features. This finding implies that additional features or more complex modeling techniques may be needed to improve classification accuracy.

### 2.3. The Performance of the Weighted Neural Network Model in Predicting LNM

**Figure 4:**
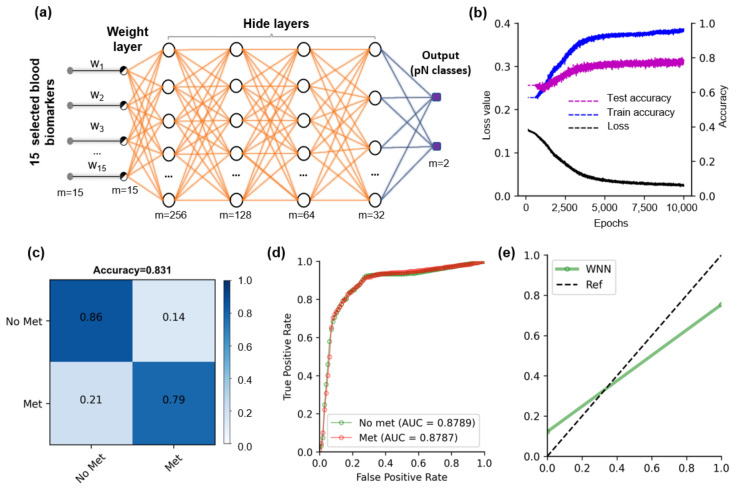
The design and performance of the WNN (weighted neural network) model for diagnosing LNM. (**a**) Structure of the WNN model. (**b**) Training loss, training accuracy, and test accuracy over training epochs. Early stopping (patience = 20 epochs) triggered termination at about 7000 epochs for this representative fold, well before the maximum of 10,000 epochs. (**c**) Confusion matrix of the WNN model for the pN classification of blood biomarkers. The classification accuracies for each class are shown in the diagonal boxes. (**d**) ROC of the WNN model for pN classification of the blood biomarkers. (**e**) Calibration curves of the WNN model, CT, and ultrasonic imaging diagnosis techniques.

To evaluate the design and performance of our WNN model for diagnosing LNM using blood biomarkers, we present multiple analyses as shown in Figure 4. In panel (a), we illustrate the structure of our WNN model, which processes 15 blood biomarkers through weighted and hidden layers to classify patients into two pN stages. Panel (b) tracks the training process, showing how accuracy improves while loss decreases over training steps. In panel (c), we display the confusion matrix, revealing an overall classification accuracy of 83.1%, with 0.86% accuracy for non-metastatic cases and 0.79% for metastatic cases. Panel (d) presents the ROC, where both classes achieve an AUC of about 0.88, demonstrating strong model performance. Finally, in panel (e), we compare the calibration curves of our WNN model with CT and ultrasound (UltraS) techniques, showing that our model achieves superior predictive accuracy. These results demonstrate that our WNN model achieves higher accuracy than conventional imaging methods in this retrospective cohort.

To assess the robustness of our model and mitigate concerns about overfitting from a single train/test split, we performed three-fold stratified cross-validation. As shown in Table S1, across the three folds, the WNN model achieved a mean accuracy of 85.0% (SD 1.5%) and a mean AUC of 0.90 (SD 0.0075), demonstrating consistent performance across different training subsets. These cross-validation metrics are closely aligned with the original test set performance, 83.1% accuracy, and an AUC of 0.88.

**Figure 5:**
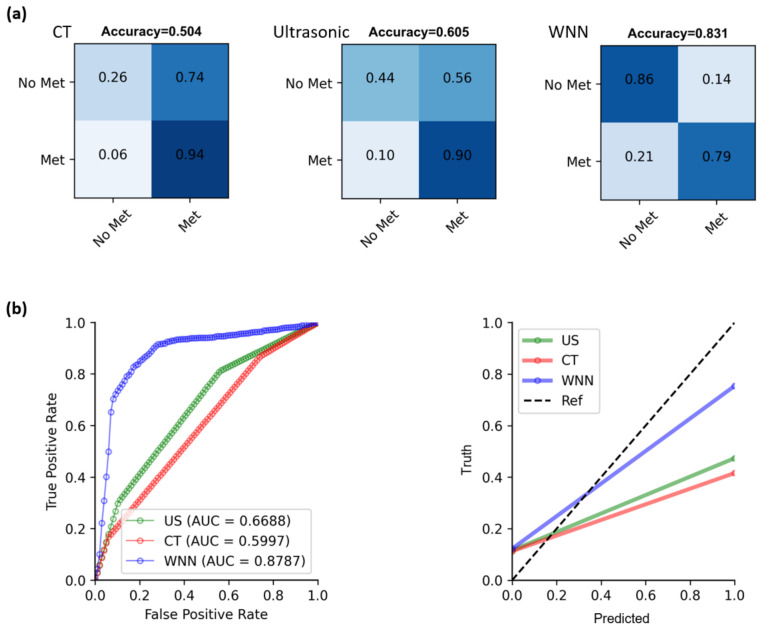
Performance comparison of diagnosing LNM into two levels (pN0 and pN1~3) using ultrasound, CT, and our WNN model. (**a**) Confusion matrix for identifying lymph node metastases (LNM). The classification accuracies for each class are shown in the diagonal boxes. (**b**) ROC for identifying LNM. Right: Calibration curve.

To compare the performance of WNN with CT and ultrasound in diagnosing LNM, we present confusion matrices, ROCs, and calibration curves as shown in Figure 5. In panel (a), we show the performance of CT, where the confusion matrix indicates an overall accuracy of 50.4%, with 94% accuracy for metastatic cases but only 26% for non-metastatic cases. The ROC reveals an AUC of about 0.60, suggesting a limited diagnostic capability. We evaluate ultrasound, which achieves a slightly higher accuracy of 60.5%, with 44% accuracy for non-metastatic cases and 90% for metastatic cases. However, the ROC shows an AUC of 0.67, indicating poor classification performance. The calibration curves for both modalities demonstrate a lack of reliable predictive power, as shown in Figure 5b. These findings highlight the limitations of CT and ultrasound in accurately diagnosing LNM, emphasizing the need for effective diagnostic methods.

### 2.4. Analysis of Impact on WNN Model Output Magnitude

**Figure 6:**
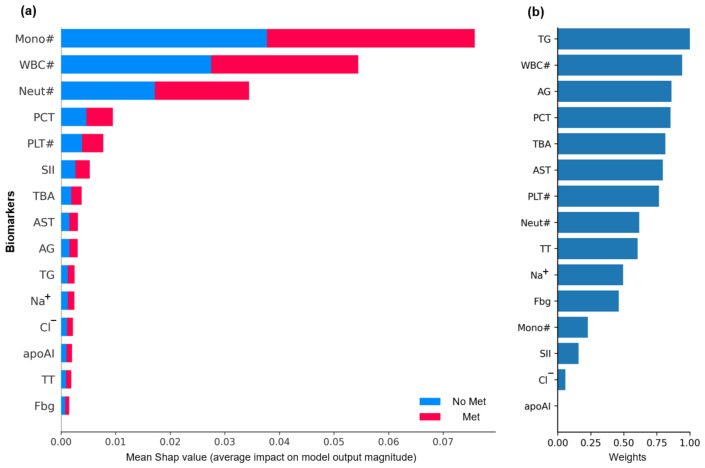
Analysis of the impact on the WNN Model output magnitude using SHAP values and the weight value of the WNN. (**a**) Illustration of blood biomarkers contributing to the pN stage of lymph node metastases using SHAP values. (**b**) The relative weights of each blood biomarker predicted using normalized values of the first layer in the WNN model.

To analyze the impact of blood biomarkers on the WNN model’s output magnitude, we present SHAP values and weight distributions in Figure 6. Panel (a) illustrates the contribution of various blood biomarkers to the prediction of LNM (pN stage) using SHAP values, where Mono#, WBC# and Neut# show the strongest influence, followed by PLT# and PCT. The color-coded bars indicate the impact direction for each class. Panel (b) displays the relative weights of blood biomarkers in the first layer of the WNN model, with TG, WBC#, AG and PCT having the highest normalized weight values. This analysis highlights the key biomarkers influencing the WNN model’s predictions, aiding in understanding the factors contributing to LNM classification.

The WNN explanation system can adopt a “dual explanation” architecture. The WNN is responsible for outputting risk probabilities while simultaneously extracting high-frequency “feature-pattern-risk” association rules (e.g., via decision trees or FP-growth). For new patients, the system calculates the match between the feature vector and the rules, generating a textual explanation based on the most relevant rule. Additionally, interactive partial dependency graphs can be generated on demand to display the positions of different key features within the model’s response surface, providing a visual representation of their combined effects. Conventional SHAP analysis struggles to detect biases related to specific geographic regions or socioeconomic groups. Therefore, it is essential to extend SHAP to assess equity across subgroups in order to avoid exacerbating social inequalities in the allocation of healthcare resources. It is necessary to further define sensitive characteristics (such as health insurance type and place of residence), calculate model performance and the average SHAP distribution of features for each subgroup, and quantify bias using measures such as the demographic parity ratio. If significant differences are detected, they can be calibrated using Platt scaling by subgroup.

### 2.5. Clinical Thresholds and Model Calibration

Firstly, to facilitate clinical adoption, we identified actionable probability thresholds for the WNN model’s LNM prediction as shown in Table S2. By using the testing set, we derived a rule-out threshold of 0.127, which achieved a sensitivity of about 91%. At this threshold, only 10% of LNM-positive patients would be missed, making it suitable for safely excluding LNM and avoiding unnecessary invasive staging. A rule-in threshold of 0.832 achieved a specificity of about 92%. Patients above this threshold may be candidates for neoadjuvant therapy or more intensive staging. The optimal Youden index threshold was 0.38, yielding a balanced sensitivity of 82.3% and specificity of 82.3%. These thresholds are summarized in Table S2.

In addition, we evaluated the calibration of the WNN model’s predicted probabilities using three metrics on the testing set, as shown in Figure S3. The Brier score was 0.1361, indicating good overall calibration (0 = perfect, 0.25 = uninformative). The calibration intercept was 0.053, close to the ideal value of 0, suggesting no systematic overestimation or underestimation of risk. The calibration slope was 0.756, indicating that the predicted probabilities are well-calibrated across the risk spectrum.

## 3. Conclusions and Discussion

Accurate lymph node status is essential for therapeutic decision-making, as it can significantly influence the choice of endoscopic mucosal or submucosal resection, the adoption of preoperative chemo(radio)therapy, and the avoidance of excessive lymphadenectomy, and improve overall mortality and morbidity of ESCC patients [18,19,20].

The best method for evaluating lymph node status in ESCC patients is lymphadenectomy after a pathological diagnosis, yet performing a comprehensive lymph node dissection via esophagectomy with radical lymphadenectomy is technically demanding and increases the risk of cardiopulmonary complications [21,22]. Therefore, there is a pressing demand for non-invasive and precise techniques to determine the LNM status in clinical applications. Non-invasive tools like CT, FDG-PET, and EUS are often utilized for diagnosing LNM; however, their diagnostic performance is not satisfactory [23]. The detection of LNM via CT is primarily based on its size, and its sensitivity is insufficient unless the LNs are larger than 10 mm [24,25]. FDG-PET can only detect tumors with a diameter exceeding 6 mm or an area over 33 mm. By integrating radiomics and deep learning features from PET/CT imaging with clinical features, there is remarkable performance (AUC of 0.955 and 0.916) in predicting cervical LNM in ESCC patients [24]. But the low spatial resolution of FDG-PET makes it challenging to separate lymph nodes close to the tumor from the tumor itself [26]. Furthermore, PET/CT examinations are expensive and not suitable for follow-up monitoring. EUS paired with fine-needle aspiration enhances the sensitivity of LNM diagnosis from 84.7% to 96.7%. However, it is only capable of evaluating LNs close to the esophagus and gastric wall [27,28].

The WNN could be triggered at specific points in the existing diagnostic workflow, rather than being applied to “all suspected patients” [29]: (1) following initial blood examination—when a patient undergoes a complete blood count due to suspected ESCC-related symptoms, the system automatically triggers the WNN calculation and returns to the electronic health record (EHR); (2) following abnormal imaging results—some patients are diagnosed with ESCC following a CT or ultrasound examination. In such cases, the WNN model can serve as a supplementary trigger to enrich the basis for risk stratification; (3) clinician-on-demand access before treatment planning—at critical points (such as before determining a treatment strategy), clinicians can proactively retrieve the WNN score as the reference information. In addition, WNN scores should be presented in a clear and concise manner on the “Patient Overview” or “Risk Assessment” panel within the EHR. The output should include clinically interpretable descriptions along with recommendations for further medical consultation. In addition, the setting of risk thresholds must accommodate both clinical feasibility and diagnostic accuracy, while also considering the costs of misclassification in different clinical scenarios. For moderate-risk cases (40–80), it is recommended to perform a complete endoscopic ultrasound and contrast-enhanced CT to confirm the presence of early-stage lesions amenable to local treatment. For high-risk cases (≥80): Trigger more aggressive clinical actions, such as upgraded examinations, initiating multidisciplinary consultations to discuss neoadjuvant therapy, or referring patients to higher-level centers.

Notably, statistical models based on clinicopathological characteristics remain the mainstream approach for LNM prediction in ESCC. Many studies have been dedicated to developing nomograms to stratify the risk of LNM in patients with early-stage and superficial ESCC [9,30]. For example, several specialized predictive models have been established specifically for T1-stage ESCC, providing valuable references for clinical decision-making [31]. Concurrently, researchers have extensively investigated the prognostic implications of lymphovascular invasion (LVI), skip LNM, and LNM classification based on lymph node stations, with these factors being incorporated into predictive models to enhance their performance [32,33]. However, a major limitation of most existing models is their reliance on postoperative pathological data or clinical features; the high-risk factors identified (e.g., tumor invasion depth, histological differentiation) are not applicable for preoperative early warning, which restricts their clinical utility in guiding preoperative therapeutic stratification.

Therefore, it is crucial to explore a minimally invasive, efficient, and easily accessible predictive method. Models based on laboratory blood tests, characterized by their convenience and potential to reflect tumor biological behaviors, are gradually becoming a research focus. Blood markers such as neutrophils, platelets, monocytes, CRP, and AST are linked to enhanced tumor growth, invasion, and angiogenesis, and can help assess tumor progression [34,35]. Alterations in the ratios of these biomarkers might indicate wider shifts in the tumor microenvironment and could be used to predict LNM in different types of cancer [15,36,37].

Machine learning (ML) and artificial intelligence (AI) technologies have emerged as powerful tools with considerable value in the identification of LNM in ESCC, offering advantages in handling high-dimensional data and improving predictive accuracy compared to traditional statistical methods [38,39]. Specifically, for T1-stage ESCC, ML models have demonstrated robust performance in predicting LNM risk, offering valuable actionable evidence to support clinical decision-making and optimize treatment strategies. Currently, the majority of research in this domain has focused on multimodal data fusion—including the integration of radiomic features, deep learning-derived characteristics, and clinicopathological parameters—as well as risk stratification for early-stage ESCC [23,40,41]. Importantly, there remains a notable research gap: ML models solely based on blood-derived parameters are relatively scarce, highlighting an unmet need for the development of non-invasive, blood-based ML tools for ESCC LNM prediction.

Numerous studies have shown that simple neural networks or ensemble methods applied to complete blood count (CBC) data can achieve robust prognostic predictions across various solid tumors. Wang et al. [42]. incorporated 25 parameters across four dimensions and used ten machine learning algorithms to construct a prognostic prediction model for non-small cell lung cancer. Among these, the neural network model performed best in predicting survival status, achieving an AUC of 0.994 and an accuracy of 0.993. Woerner et al. [43]. used automated machine learning methods to construct a colorectal cancer prognostic model and successfully identified which stage IIA patients would benefit from adjuvant chemotherapy. In breast cancer, machine learning models based on complete blood counts have been used for risk stratification within six months, demonstrating the potential of complete blood counts as a risk stratification tool [44]. In various solid tumors, including lung, gastric, and breast cancers, machine learning models have demonstrated the ability to extract prognostic information from routine blood tests, inflammatory markers, and nutritional indicators. This highlights a growing trend in clinical oncology: combining routine laboratory data with machine learning to develop low-cost, scalable tools for cancer prognosis prediction.

Against this backdrop, we screened 66 features from 95 regular laboratory tests to build the novel WNN model for accurate identification of LNM in patients with ESCC. Our model utilizing the WNN classifier demonstrated high performance in both internal and external testing cohorts. The WNN model achieved an overall accuracy of 83.1% for diagnosing LNM based on blood biomarkers, which outperforms conventional imaging methods of CT and ultrasound (50.4% and 60.5%). It has been documented that blood biomarkers serve as predictors for LNM in ESCC. In a retrospective study by Ohsawa et al., 140 out of 185 patients with cT1N0M0 ESCC (75.7%) had pN0 status, and the AUC values for NLR, PLR, and LMR were 0.8897, 0.7241, and 0.7018, respectively, for predicting pathological LNM [15]. Our innovative model showed impressive diagnostic precision, indicating that the identification of LNM using routine blood tests is feasible and could be applied in clinical settings. Moreover, our model utilizes routine blood test parameters, making it both economical and simple to generalize. Furthermore, multiple studies have confirmed that various hematological biomarkers can predict preoperative LNM in different cancers. For instance, fibrinogen and monocyte-to-lymphocyte ratio (MLR) can predict LN metastasis in patients with high-grade T1 urothelial carcinoma, while lipoprotein(a) is effective for assessing the risk of non-sentinel LNM in Chinese patients with breast cancer [45,46]. These results solidify the validity of blood-based predictive models and highlight their broad application potential. Future studies may further explore the clinical utility of such models for LNM evaluation in other cancer types.

Despite these promising findings, this study has several limitations that should be acknowledged. First, this was a single-center study, and the generalizability of the WNN model thus requires further validation in multi-center cohorts to mitigate selection bias and enhance robustness. External validation should be conducted using datasets from different geographic regions and healthcare facilities (at least 3–4 independent centers), assessing the stability and discrimination of the WNN model. In addition, prospective clinical trials can be designed to integrate the WNN into diagnostic workflows, using specific metrics (such as change rate in physician decisions or staging accuracy) as primary endpoints. Finally, in practical clinical applications, an automated monitoring dashboard can be established to periodically review and update. It is favorable to facilitate the safe, effective, and sustainable clinical application of this WNN model.

Second, all samples were recruited exclusively from Chinese populations, which may limit the extrapolation of results to other ethnicities or regions—blood test results can vary across populations due to differences in genetic backgrounds, dietary habits, environmental exposures, and laboratory standards, potentially affecting model performance in non-Chinese groups.

Currently, WNN models still present critical challenges in clinical applications. (1) Standardization of blood biomarker measurements: Methodological differences among laboratories for the same indicator can lead to bias in the distribution of input features, which requires the establishment of calibration mapping functions or uniform testing protocols. (2) Missing data handling: Multiple interpolation or missing-value-aware algorithms can be implemented, and WNN scores should not be generated when the missing data exceeds a threshold. (3) Model updates over time: Due to concept drift (changes in population characteristics and treatment regimens), the model must maintain performance through periodic retraining and incremental learning. (4) Regulatory approval for WNN medical devices: Since WNN’s clinical decision support system outputs can influence treatment decisions, they are typically classified as Class III medical devices and must undergo clinical trials that meet established standards.

## 4. Methods in Detail

### 4.1. Patients and Study Design

This retrospective study from a large cancer hospital in China, Sun Yat-sen University Cancer Center, Guangzhou (SYSUCC cohort, *n* = 1933). All ESCC participants underwent pathological confirmation for the presence or absence of LN metastasis. Laboratory tests taken within one month before diagnosis to the start of any treatment were considered. Work with human subjects was approved by the Institutional Review Board of Sun Yat-sen University Cancer Center.

### 4.2. Laboratory Examination

We screened 66 indicators from 95 candidates. Parameters with a patient detection rate greater than 95% were included (Supplementary Table S4).

Complete blood count results were obtained using the automated hematology analyzer XN-2000 (Sysmex Corporation, Kobe, Japan), including RBC, WBC (percentage and absolute number of neutrophils, lymphocytes, eosinophils, basophils, and monocytes), mean corpuscular volume (MCV), hematocrit (HCT), platelets (PLT), hemoglobin levels (HGB), mean corpuscular hemoglobin concentration (MCHC), and mean corpuscular hemoglobin (MCH).

Biochemical parameters were measured on a Roche Cobas C702 Chemistry Analyzer from Roche Diagnostics (Tokyo, Japan) and a Hitachi LABOSPECT 008 AS Chemistry Analyzer from Hitachi High-Tech Corporation (Tokyo, Japan). Using automated procedures: albumin (ALB), alkaline phosphatase (ALP), alanine aminotransferase (ALT), aspartate aminotransferase (AST), apolipoprotein A1 (ApoA1), apolipoprotein B (ApoB), calcium (Ca+), cholinesterase (CHE), total cholesterol (CHO), creatine kinase (CK), carbon dioxide (CO2), creatinine (CRE), C-reaction protein (CRP), cystatin C (CYSC), glucose (GLU), urea, uric acid (UA), total bile acid (TBA), triglycerides (TG), gamma-glutamyl transferase (GGT), total proteins (TP), globulin (GLOB), lactate dehydrogenase (LDH), total bilirubin (total bilirubin (TBA), direct bilirubin (DBIL), high-density lipoprotein-C (HDL-C), low-density lipoprotein-C (LDL-C), serum amyloid (SAA), activated partial thromboplastin time (APTT), prothrombin time (PT), fibrinogen (FBG), thrombin time (TT), and D-dimer (D-Dimer) were analyzed using the Sysmex CA-7000 coagulation analyzer (Sysmex Corporation, Kobe, Japan). NLR, MLR, PLR, and Systemic Immune-Inflammation Index (SII) were calculated as ratios of neutrophil count/lymphocyte count (NLR), monocyte count/lymphocyte count (MLR), platelet count/lymphocyte count (PLR), and NLR * platelet count, respectively.

For each candidate Biomarker, we performed the Wilcoxon Rank Sum test to compare distributions between the pN0 and pN1-3 groups within the training set. Features with “*p*” < 0.01 were retained. This pre-specified threshold was chosen to reduce false positives while preserving potentially relevant biomarkers.

### 4.3. Establishment of Neural Network Model

The WNN model consisted of four layers: one input layer and three hidden layers. The input layer had 15 nodes (m = 15), which correspond to the 15 selected biomarkers. The node number in the 1st, 2nd, 3rd and 4th hiding layer was 256 (m = 256), 128 (m = 128), 64 (m = 64) and 32 (m = 32), respectively. The details of the WNN model are described in Table S3. The term “weighted” in our WNN model refers to the learnable weighting mechanisms, both optimized end-to-end during training without manual assignment. Before entering the first hidden layer, each of the 15 normalized input biomarkers *x_i_* is multiplied by a learnable feature weight *w_f,i_* (initialized to 1.0), yielding a weighted input vector *x_wi_* = *x_i_*_×_ *w_f,i_*. This mechanism allows the network to adaptively emphasize biomarkers with greater predictive relevance, as guided by backpropagation. The weighting mechanisms are jointly optimized during training. The network architecture (from “input layer” to “three hidden layers of 256, 128, and 64 nodes” to “output layer”) follows a standard fully connected topology with ReLU activation and dropout regularization.

Before training, the original data of blood biomarkers was normalized in a range from 0 to 1. The training dataset was enhanced by adding Gaussian noise with an SNR of 40 dBm. In the training of a multi-label network, each input had one label (Figure 4a). The label represented the pN type labeled as a binary vector with one element being 1 and the others being zero. To avoid vanishing gradients and achieve fast computation, we adopted a ReLU function as the activation function for each node in the hidden layers. The WNN was trained for over 10,000 epochs using the Adam optimizer with a learning rate set at 0.0005. The SYSUCC cohort was used for training and validation. We have acquired 1933 original samples, and 1121 (≈58%) randomly selected from the samples were used in training the WNN. The 812 (≈42%) samples were used in the test of the WNN. The performance of classification and prediction with the WNN was evaluated by the receiver operating curve (ROC) and the correlation analysis. To test the algorithm’s sensitivity to noise levels, the relationship between noise percentage and network performance was investigated.

### 4.4. CT and Endoscopic Ultrasonography

All CT and endoscopic ultrasonography (EUS) data in this retrospective study were extracted from archived routine clinical reports. Each imaging report was independently evaluated by two radiologists, and one of them was a senior physician with abundant diagnostic experience. Lymph node status on CT was graded as 0 (no LNM), 1 (reactive lymph nodes), 2 (LN, undetermined), and 3 (suspicious LNM). EUS was interpreted using the standard uN staging system (grades 0, 1, 2, 3).

We enrolled patients whose pretreatment CT and EUS examinations were completed at our center. Patients with luminal stenosis that prevented full endoscopic assessment of lymph node status were excluded. Only patients with full preoperative CT and EUS data were included for comparison with the WNN model.

## Figures and Tables

**Table 1:** Clinical characteristics of patients in the cohort.

Items	Training Cohort		Testing Cohort		*p*-Value
Age (Average ± SD)	66.5 ± 8.5		64.7 ± 7.9		-
Gender	Male (890)	Female (231)	Male (622)	Female (190)	0.294
pN Stage	pN0 (622)	pN1-3 (499)	pN0 (520)	pN1-3 (292)	-

## Data Availability

The raw data supporting the conclusions of this article will be made available by the authors on request.

## Supplementary Materials

The following supporting information can be downloaded at https://www.mdpi.com/article/10.3390/bios16070377/s1, Figure S1: The blood biomarkers that have no significant impact on pN stage of lymph node metastases with significance > 0.01. *p* in bracket represents the *p*-value of difference significance analysis between a biomarker value of Metastases and without Metastases. The significance analysis is carried by Wilcoxon Rank Sum tests for any of the biomarkers.; Figure S2: Performance comparison of diagnosing LNM into four levels (pN0, pN1, pN2 and pN3) using Ultrasound, CT and our WNN model. Figure S3: Cut-off and calibration analysis of the WNN model; Table S1: The results of 3-fold cross-validation; Table S2: Clinically actionable thresholds for LNM prediction using the WNN model. Table S3: Full specification of the Weighted Neural Network (WNN) model. Table S4: List of laboratory parameters with full names and units.
