# Supplementary material for: A Weighted Neural Network Model Based on Laboratory Tests for Identifying Lymph Node Metastases in Esophageal Squamous Cell Carcinomas
Source: Biosensors (Basel). 2026 Jul 10;16(7):377. doi: 10.3390/bios16070377 (PMC13406964; doi:10.3390/bios16070377)
Supplement: Supplementary file 1 [file biosensors-16-00377-s001.zip › biosensors-4312170-supplementary.pdf]

A Weighted Neural Network Model based on laboratory tests for identifying lymph node metastases in esophageal squamous cell carcinomas

Qiangqiang Ouyang[1#], Ziming Gao[1], Jingbo Yang[1#], Shaoyi Wang[2#], Zonglin Li[1], Yifan Zhang[1], Tianyou Chen[1], Xinhua Xu[3], Runkun Han[3*], and Hao Chen[3*]

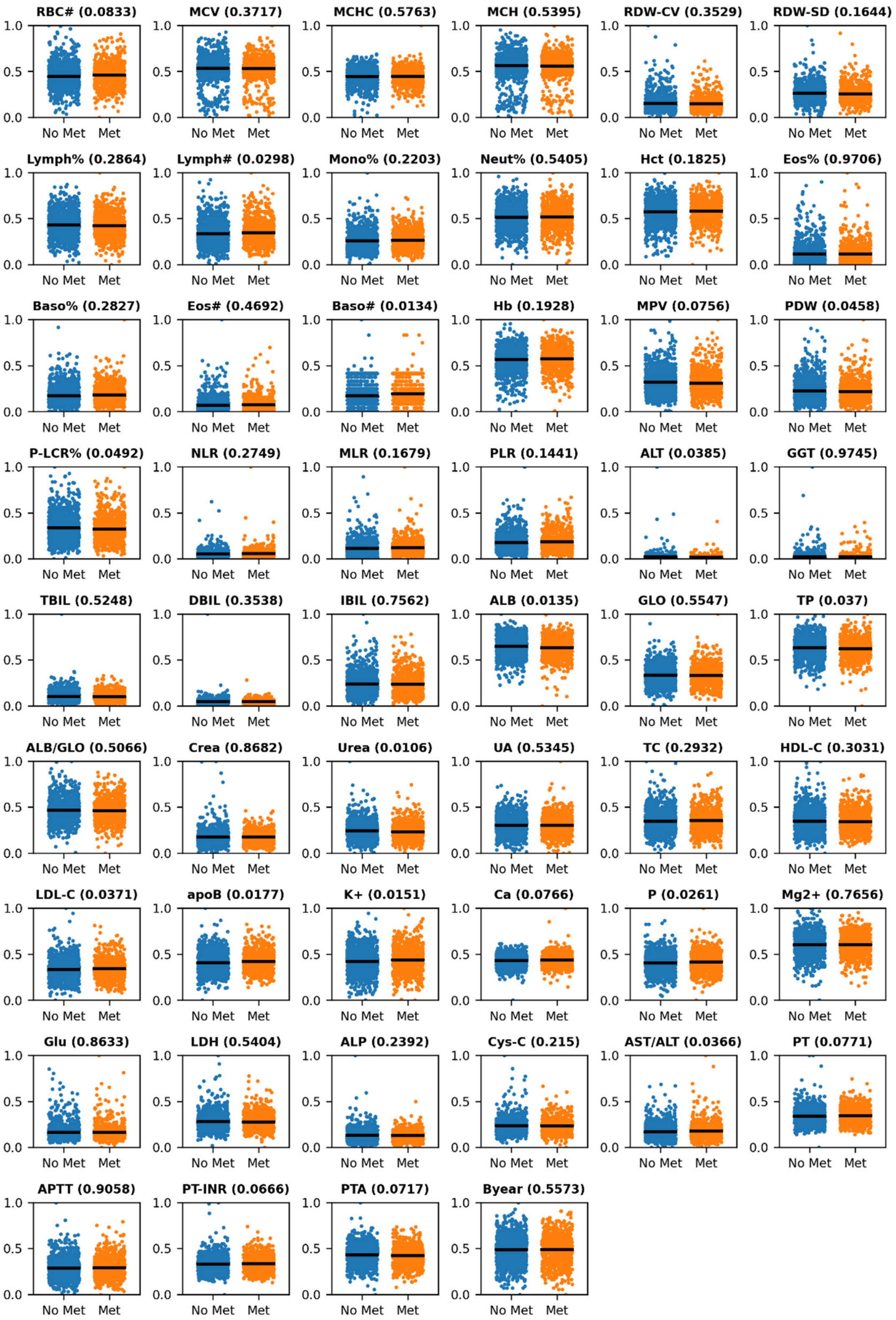

Figure S1. The blood biomarkers that have not significant impact on pN stage of lymph node metastases with significance >0.01. p in bracket represents the p-value of difference significance analysis between a biomarker value of Metastases and without Metastases. The significance analysis is carried by Wilcoxon Rank Sum tests for any of the biomarkers.

Supplementary Table S1 The results of 3-fold cross-validation

Items	Accuracy 2 classes	AUC 2 classes	Accuracy 4 classes	AUC 4 classes
Trial-1	0.8292	0.8922	0.7842	0.8837
Trial-2	0.8574	0.9015	0.7953	0.8937
Trial-3	0.8634	0.9105	0.7966	0.9226
Mean value	**0.8500**	**0.9014**	0.7920	0.9000
Standard deviation	**0.0149**	**0.0075**	0.0056	0.0165

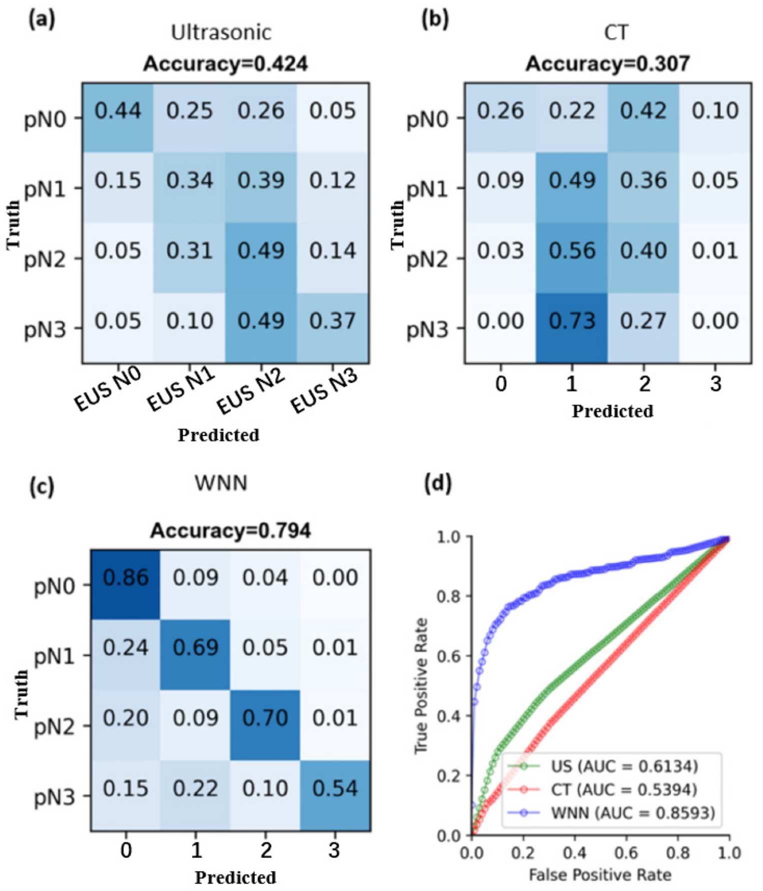

Figure S2. Performance comparison of diagnosing LNM into four levels (pN0, pN1, pN2 and pN3) using Ultrasound, CT and our WNN model. (a-c) Confusion matrix for identifying Lymph Node Metastases (LNM) using Ultrasound, CT and our WNN model. The classification accuracies for each class are shown in the diagonal boxes. (d) ROC curve for identifying LNM. Right: Calibration curve.

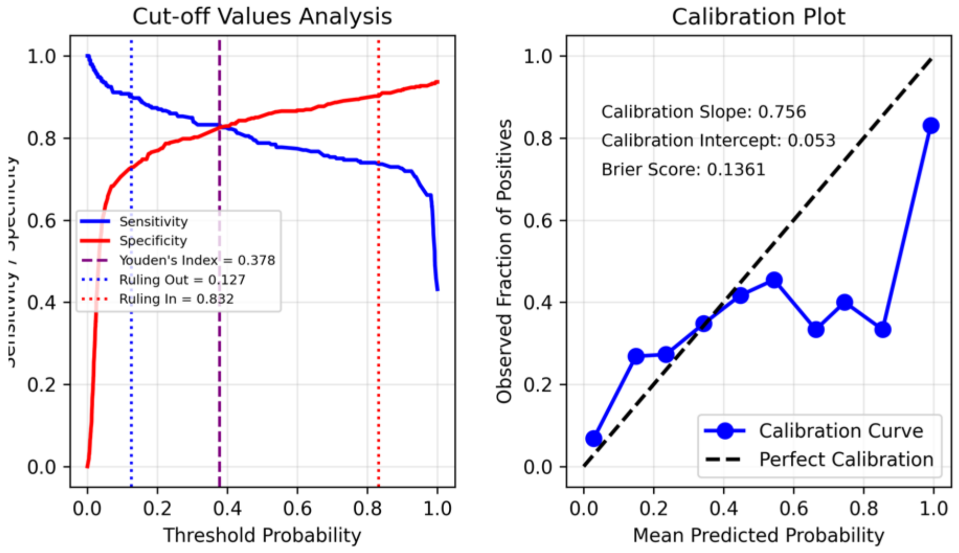

Figure S3. Cut-off and calibration analysis of the WNN model

Supplementary Table S2. Clinically actionable thresholds for LNM prediction using the WNN model.

Threshold Type	Cut-off Value	Sensitivity (%)	Specificity (%)	Clinical Action
Rule-out	0.127	≈91%	≈76%	low risk: avoid or delay invasive staging (e.g., EUS-FNA, surgery)
Rule-in	0.832	≈78%	≈92%	high risk: recommend neoadjuvant therapy or advanced imaging (PET-CT)
Optimal Youden	0.38	≈82%	≈82%	intermediate risk: general reference (balances missed vs. unnecessary treatment)

Supplementary Table S3. Full specification of the Weighted Neural Network (WNN) model.

Category	Parameter	Value
Architecture	Input layer nodes	15
	Hidden layer 1 nodes	256
	Hidden layer 2 nodes	128
	Hidden layer 3 nodes	64
	Hidden layer 4 nodes	32
	Output layer activation	RELU
	Dropout rate (after each hidden layer)	0.2
Loss function	Type	MSELoss
Optimizer	Type	Adam
	Learning rate	1e-4
Regularization	L2 weight decay	2e-4
Training	Batch size	Full size (1121)
	Max epochs	10,000 (early stopping applied)
	Test split	56% of training set
	Data augmentation	Gaussian noise, SNR = 40 dBm
	Augmented samples per epoch	13,531

Supplementary Table S4. List of laboratory parameters with full names and units

Abbreviation	Full Name	Unit	Included in 66 screened features	Included in 15 final model biomarkers
WBC#	White blood cell count	$\times 10^9/L$	☑ Yes / □ No	☑ Yes
RBC#	Red blood cell count	$\times 10^{12}/L$	☑ Yes / □ No	
Hb	Hemoglobin	g/L	☑ Yes / □ No	
Hct	Hematocrit	%	☑ Yes / □ No	
MCV	Mean corpuscular volume	fL	☑ Yes / □ No	
MCH	Mean corpuscular hemoglobin	pg	☑ Yes / □ No	
MCHC	Mean corpuscular hemoglobin concentration	g/L	☑ Yes / □ No	
RDW-CV	Red cell distribution width-coefficient of variation	%	☑ Yes / □ No	
RDW-SD	Red cell distribution width-standard deviation	fL	☑ Yes / □ No	
Lymph%	Lymphocyte percentage	%	☑ Yes / □ No	
Lymph#	Lymphocyte absolute count	$\times 10^9/L$	☑ Yes / □ No	
Mono%	Monocyte percentage	%	☑ Yes / □ No	
Mono#	Monocyte absolute count	$\times 10^9/L$	☑ Yes / □ No	☑ Yes
Neut%	Neutrophil percentage	%	☑ Yes / □ No	

Abbreviation	Full Name	Unit	Included in 66 screened features	Included in 15 final model biomarkers
Neut#	Neutrophil absolute count	×10⁹/L	☑ Yes / □ No	☑ Yes
Eos%	Eosinophil percentage	%	☑ Yes / □ No	
Eos#	Eosinophil absolute count	×10⁹/L	☑ Yes / □ No	
Baso%	Basophil percentage	%	☑ Yes / □ No	
Baso#	Basophil absolute count	×10⁹/L	☑ Yes / □ No	
PLT#	Platelet count	×10⁹/L	☑ Yes / □ No	☑ Yes
MPV	Mean platelet volume	fL	☑ Yes / □ No	
PDW	Platelet distribution width	%	☑ Yes / □ No	
PCT	Plateletcrit	%	☑ Yes / □ No	☑ Yes
P-LCR%	Platelet large cell ratio	%	☑ Yes / □ No	
NRBC#	Nucleated red blood cell absolute count	×10⁹/L	□ Yes / ☑ No	
NRBC%	Nucleated red blood cell percentage	%	□ Yes / ☑ No	
IG#	Immature granulocyte absolute count	×10⁹/L	□ Yes / ☑ No	
IG%	Immature granulocyte percentage	%	□ Yes / ☑ No	
NLR	Neutrophil-to-lymphocyte ratio	No unit	☑ Yes / □ No	

Abbreviation	Full Name	Unit	Included in 66 screened features	Included in 15 final model biomarkers
MLR	Monocyte-to-lymphocyte ratio	No unit	☑ Yes / □ No	
PLR	Platelet-to-lymphocyte ratio	No unit	☑ Yes / □ No	
SII	Systemic immune-inflammation index	No unit	☑ Yes / □ No	☑ Yes
ALT	Alanine aminotransferase	U/L	☑ Yes / □ No	
AST	Aspartate aminotransferase	U/L	☑ Yes / □ No	☑ Yes
AST/ALT	AST/ALT ratio	No unit	☑ Yes / □ No	
GGT	Gamma-glutamyltransferase	U/L	☑ Yes / □ No	
ALP	Alkaline phosphatase	U/L	☑ Yes / □ No	
ChE	Cholinesterase	U/L	□Yes / ☑ No	
TBIL	Total bilirubin	μmol/L	☑ Yes / □ No	
DBIL	Direct bilirubin	μmol/L	☑ Yes / □ No	
IBIL	Indirect bilirubin	μmol/L	☑ Yes / □ No	
TP	Total serum protein	g/L	☑ Yes / □ No	
ALB	Albumin	g/L	☑ Yes / □ No	
GLO	Globulin	g/L	☑ Yes / □ No	
ALB/GLO	Albumin/globulin ratio	No unit	☑ Yes / □ No	
TBA	Total bile acid	μmol/L	☑ Yes / □ No	☑ Yes
Crea	Serum creatinine	μmol/L	☑ Yes / □ No	
Urea	Urea	mmol/L	☑ Yes / □ No	

Abbreviation	Full Name	Unit	Included in 66 screened features	Included in 15 final model biomarkers
UA	Uric acid	μmol/L	☑ Yes / □ No	
Cys-C	Cystatin C	mg/L	☑ Yes / □ No	
TC	Total cholesterol	mmol/L	☑ Yes / □ No	
TG	Triglyceride	mmol/L	☑ Yes / □ No	☑ Yes
HDL-C	High-density lipoprotein cholesterol	mmol/L	☑ Yes / □ No	
LDL-C	Low-density lipoprotein cholesterol	mmol/L	☑ Yes / □ No	
apoAI	Apolipoprotein A-I	g/L	☑ Yes / □ No	☑ Yes
apoB	Apolipoprotein B	g/L	☑ Yes / □ No	
K^+	Potassium	mmol/L	☑ Yes / □ No	
Na^+	Sodium	mmol/L	☑ Yes / □ No	☑ Yes
Cl^-	Chloride	mmol/L	☑ Yes / □ No	☑ Yes
Ca	Calcium	mmol/L	☑ Yes / □ No	
P	Inorganic phosphorus	mmol/L	☑ Yes / □ No	
Mg^{2+}	Magnesium	mmol/L	☑ Yes / □ No	
AG	Anion gap	No unit	☑ Yes / □ No	☑ Yes
Glu	Glucose	mmol/L	☑ Yes / □ No	
LDH	Lactate dehydrogenase	U/L	☑ Yes / □ No	
PT	Prothrombin time	s	☑ Yes / □ No	
PT-INR	Prothrombin time-international normalized ratio	No unit	☑ Yes / □ No	

Abbreviation	Full Name	Unit	Included in 66 screened features	Included in 15 final model biomarkers
APTT	Activated partial thromboplastin time	s	☑ Yes / □ No	
TT	Thrombin time	s	☑ Yes / □ No	☑ Yes
Fbg	Fibrinogen	g/L	☑ Yes / □ No	☑ Yes
PTA	Prothrombin activity	%	☑ Yes / □ No	
D-Dimer	D-dimer	μg/mL	□ Yes / ☑ No	
FDP	Fibrinogen and fibrin degradation products	μg/mL	□ Yes / ☑ No	
AFP	Alpha-fetoprotein	ng/mL	□ Yes / ☑ No	
CEA	Carcinoembryonic antigen	ng/mL	□ Yes / ☑ No	
SCC	Squamous cell carcinoma antigen	ng/mL	□ Yes / ☑ No	
CA199	Carbohydrate antigen 19-9	U/mL	□ Yes / ☑ No	
CA125	Carbohydrate antigen 125	U/mL	□ Yes / ☑ No	
CA724	Carbohydrate antigen 72-4	U/mL	□ Yes / ☑ No	
CA153	Carbohydrate antigen 15-3	U/mL	□ Yes / ☑ No	
TPSA	Total prostate-specific antigen	ng/mL	□ Yes / ☑ No	
FPSA	Free prostate-specific antigen	ng/mL	□ Yes / ☑ No	
FPSA/TPSA	Free-to-total prostate-specific antigen ratio	No unit	□ Yes / ☑ No	

Abbreviation	Full Name	Unit	Included in 66 screened features	Included in 15 final model biomarkers
NSE	Neuron-specific enolase	ng/mL	□ Yes / ☑ No	
CYFRA21-1	Cytokeratin 19 fragment	ng/mL	□ Yes / ☑ No	
CRP	C-reactive protein	mg/L	□ Yes / ☑ No	
SAA	Serum amyloid A	mg/L	□ Yes / ☑ No	
FT3	Free triiodothyronine	pmol/L	□ Yes / ☑ No	
FT4	Free thyroxine	pmol/L	□ Yes / ☑ No	
T3	Total triiodothyronine	nmol/L	□ Yes / ☑ No	
T4	Total thyroxine	nmol/L	□ Yes / ☑ No	
TSH	Thyroid-stimulating hormone	uIU/L	□ Yes / ☑ No	
A-TPO	Anti-thyroid peroxidase antibody	U/mL	□ Yes / ☑ No	
A-TG	Anti-thyroglobulin antibody	IU/mL	□ Yes / ☑ No	
TG	Thyroglobulin	ng/mL	□ Yes / ☑ No	
